# Clinical isolates of *Candida auris* with enhanced adherence and biofilm formation due to genomic amplification of *ALS4*

**DOI:** 10.1371/journal.ppat.1011239

**Published:** 2023-03-13

**Authors:** Jian Bing, Zhangyue Guan, Tianhong Zheng, Zhijie Zhang, Shuru Fan, Craig L. Ennis, Clarissa J. Nobile, Guanghua Huang

**Affiliations:** 1 Shanghai Institute of Infectious Disease and Biosecurity, Department of infectious diseases, Huashan Hospital and State Key Laboratory of Genetic Engineering, School of Life Sciences, Fudan University, Shanghai, China; 2 Shanghai Engineering Research Center of Industrial Microorganisms, Shanghai, China; 3 Department of Laboratory Medicine, Shengjing Hospital of China Medical University, Shenyang, China; 4 Department of Molecular and Cell Biology, University of California, Merced, California, United States of America; 5 Health Sciences Research Institute, University of California, Merced, California, United States of America; Geisel School of Medicine at Dartmouth, UNITED STATES

## Abstract

*Candida auris* is an emerging multidrug-resistant fungal pathogen and a new global threat to human health. A unique morphological feature of this fungus is its multicellular aggregating phenotype, which has been thought to be associated with defects in cell division. In this study, we report a new aggregating form of two clinical *C*. *auris* isolates with increased biofilm forming capacity due to enhanced adherence of adjacent cells and surfaces. Unlike the previously reported aggregating morphology, this new aggregating multicellular form of *C*. *auris* can become unicellular after treatment with proteinase K or trypsin. Genomic analysis demonstrated that amplification of the subtelomeric adhesin gene *ALS4* is the reason behind the strain’s enhanced adherence and biofilm forming capacities. Many clinical isolates of *C*. *auris* have variable copy numbers of *ALS4*, suggesting that this subtelomeric region exhibits instability. Global transcriptional profiling and quantitative real-time PCR assays indicated that genomic amplification of *ALS4* results in a dramatic increase in overall levels of transcription. Compared to the previously characterized nonaggregative/yeast-form and aggregative-form strains of *C*. *auris*, this new Als4-mediated aggregative-form strain of *C*. *auris* displays several unique characteristics in terms of its biofilm formation, surface colonization, and virulence.

## Author summary

The emerging fungal pathogen *Candida auris* has rapidly spread worldwide in the past several years. This fungus is considered to be a “superbug” pathogen due to its multidrug-resistant properties and high rates of transmission in clinical settings. Unlike other pathogenic species in the *Candida* genus, many clinical isolates of *C*. *auris* can form a unique morphology, namely a multicellular aggregating phenotype, that is thought to be caused by defects in cell division. In this study, we report a new aggregating form observed from two clinical isolates of *C*. *auris* with increased adhesion and biofilm forming capacity. Genomic analysis demonstrated that amplification of the subtelomeric adhesin gene *ALS4* is the reason behind the strain’s enhanced adherence and biofilm forming capacities. Compared to the previously characterized nonaggregative/yeast-form and aggregative-form strains, this new Als4-mediated aggregative-form strain of *C*. *auris* displays many unique characteristics in terms of its biofilm formation, surface colonization, and virulence.

## Introduction

The emerging fungal pathogen *Candida auris* is becoming a serious global health threat. Due to its multidrug-resistant features, invasive infections of *C*. *auris* often result in high mortality rates [1]. Like other frequently isolated and pathogenic *Candida* species in clinical settings, such as *Candida albicans*, *Candida tropicalis*, and *Candida parapsilosis* (and with the exception of *Candida glabrata*), *C*. *auris* belongs to the CTG (*Candida*) clade, where it translates the CTG codon as serine instead of leucine [2]. First described in 2009, *C*. *auris* was isolated from the external ear of a female Japanese patient [3].

*C*. *auris* is not only capable of undergoing phenotypic transitions and biofilm development [4–6], but it is also capable of forming unique multicellular aggregates [7–9]. The aggregating and nonaggregating cells of *C*. *auris* differ in a number of aspects including their abilities to form biofilms, as well as their virulence and antifungal drug resistance properties [7,10,11], According to the US Centers for Disease Control and Prevention, more than 40 countries have reported cases of *C*. *auris* colonization or infection as of 2021 [4,10–12]. Borman et al. (2016) suggested that the formation of the aggregating morphology in *C*. *auris* is associated with a defect in cell division and the failure to release daughter cells after budding [7]. In the *Galleria mellonella* infection model, the aggregating cells of *C*. *auris* showed significantly attenuated virulence relative to nonaggregating cells [7]. Two studies demonstrated that the nonaggregating isolates of *C*. *auris* developed more robust biofilms than their aggregating counterparts [4,11]; another study observed no association between *C*. *auris* cellular aggregation and the ability to form biofilms [10].

It has been reported that chromosomal rearrangements and loss of subtelomeric adhesins including genes encoding agglutinin-like sequence (Als) adhesins often occur in *C*. *auris* clinical strains [13]. The Als protein family plays critical roles in the regulation of biofilm development, adhesion to host cells and surfaces, and pathogenicity for many *Candida* species [14,15]. Als proteins typically have a three-domain structure consisting of an N-terminal immunoglobulin-like domain, a central domain with variable tandem repeats that are rich in serine and threonine, and a C-terminal domain containing a glycosylphosphatidylinositol (GPI) anchor. *ALS* genes exhibit a high level of variability among clinical isolates of *C*. *albicans*, including strain- and allele-specific gene sizes, strain-specific coding regions, differences in transcriptional regulation, and varying gene copy numbers [14,15]. This variability may confer natural *Candida* strains with the capacity to adapt to distinct host niches. In *Saccharomyces cerevisiae*, the *FLO* gene family includes a set of telomere-adjacent genes encoding cell wall glycoproteins that play similar roles to *Candida* Als proteins, including the regulation of biofilm development, flocculation, and cell-surface adhesion [16]. These *FLO* genes, which are located at subtelomeric regions, are often subject to genetic and epigenetic regulation to generate cell-surface variation in *S*. *cerevisiae* [17].

In the current study, we report a new aggregating form of *C*. *auris* with strongly enhanced biofilm forming capacity. This multicellular morphology is the result of increased adherence of adjacent cells and is not due to defects in cell division or the failure to release daughter cells. Whole genome sequencing experiments demonstrated that the genomic amplification of the cell wall adhesin gene *ALS4* is responsible for this new *C*. *auris* aggregating morphology. Further investigation indicated that *ALS4* is located at the subtelomeric region of chromosome 5 and exhibits a high level of sequence and copy number variations (CNVs) in clinical *C*. *auris* isolates.

## Results

### Isolation and identification of the nonaggregative/yeast-form and aggregative-form strains of *C*. *auris*

We isolated a *C*. *auris* nonaggregative-form (yeast-form) strain (SJ01) and an aggregative-form strain (SJ02) simultaneously from a urine sample of a long-term care female patient (aged 81), who had been admitted to the Department of Respiratory Medicine at the Shengjing Hospital of China Medical University (Shenyang, China) and had been diagnosed with pneumonia. The patient received long-term antibiotic treatment therapy for >1 year and had a history of hypertension.

The strains SJ01 and SJ02 were initially identified as *C*. *auris* by MALDI-TOF mass spectrometry and were further verified by whole genome sequencing. The two strains belong to *C*. *auris* clade III (the South African clade) and are closely related, with only 38 single-nucleotide polymorphisms (SNPs) and 90 short insertions/deletions (INDELs) in total across their genomes (**S1 Table and S1 Dataset**), indicating their close genetic relationship.

### Morphological and virulence analyses of the nonaggregative/yeast-form and aggregative-form strains of *C*. *auris*

**Fig 1:**
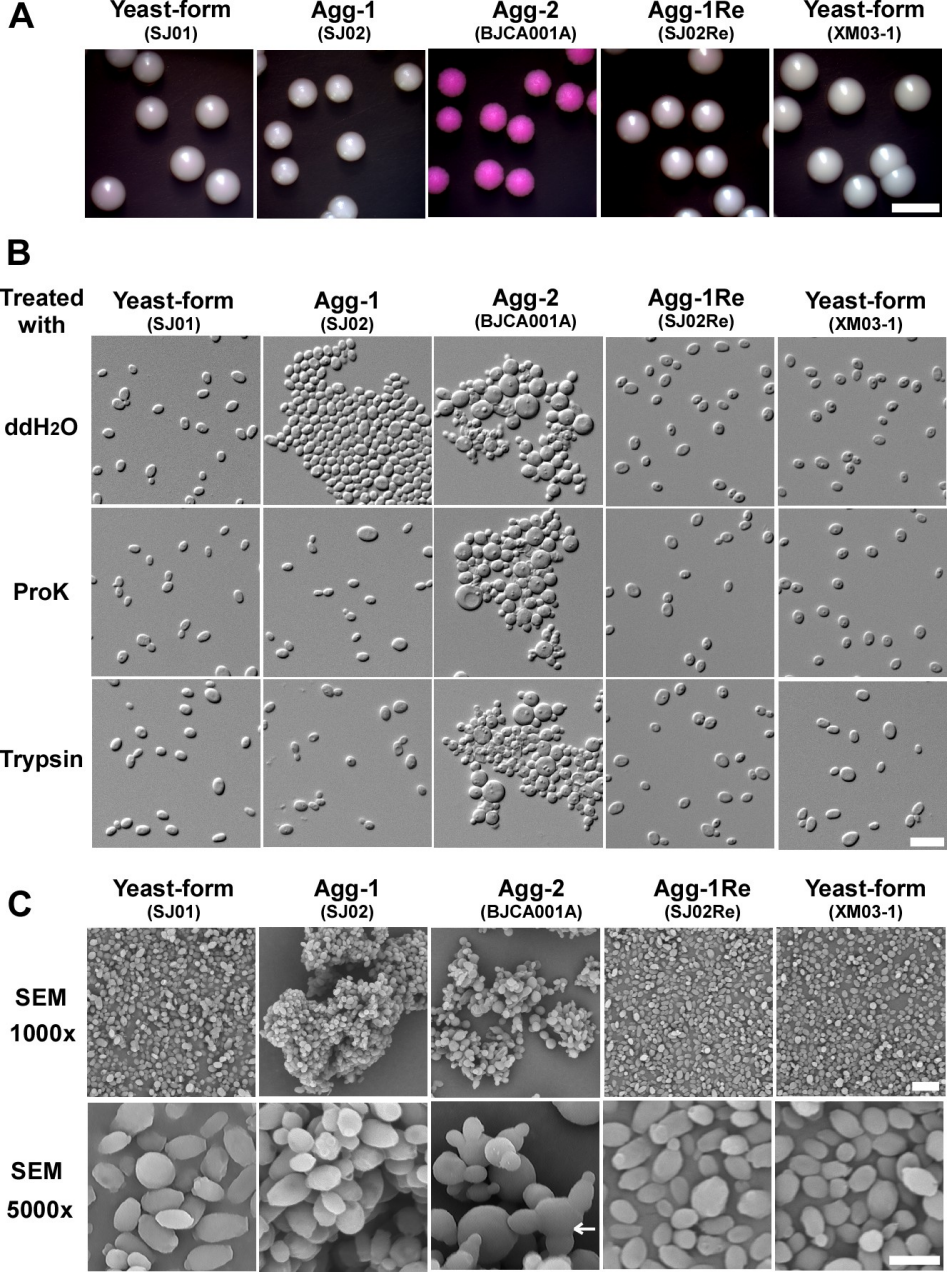
Colony and cellular morphologies of the nonaggregative/yeast-form (SJ01), Agg-1 (SJ02), Agg-2 (BJCA001A), Agg-1Re (SJ02Re), and XM03-1 strains of *C*. *auris*. Strains SJ01 and SJ02 were isolated from the same patient. BJCA001A is the aggregating form of strain BJCA001. The nonaggregative/yeast-form strain SJ02Re was spontaneously generated form cells of strain SJ02 grown on YPD medium. Strain SJ02Re carries a single copy of *ALS4*. Strain XM03-1 is a clinical isolate that is missing *ALS4*. (A) Colony morphologies. *C*. *auris* cells were plated on YPD medium containing 5 μg/mL phloxine B for 4 days at 30°C. Scale bar = 5 mm. (B) DIC images of *C*. *auris* cells treated with ddH_2_O, proteinase K, or trypsin. *C*. *auris* cells of a single colony of each strain were washed with 1 x PBS and then treated with ddH_2_O, proteinase K, or trypsin for 1 hour at 37°C. Scale bars,10 μm. (C) Low and high magnification SEM images of *C*. *auris* cells. The culture condition was the same as used in panel A. Scale bar,10 μm. Strains SJ02Re and XM03-1 served as controls.

To compare the biological features of strains SJ01 and SJ02, the strains were plated onto solid YPD rich medium containing the red dye phloxine B and incubated at 30°C for four days. Strain BJCA001A (Agg-2), a derivative of strain BJCA001, containing previously reported typical aggregative-form cells [18], which displayed a clear defect in cell division [7], served as a reference strain. As shown in **Fig 1A**, both SJ01 and SJ02 strains formed white and shiny colonies, whereas the typical aggregating isolate BJCA001A formed red and rough colonies on YPD plates containing phloxine B. We next examined the cellular morphologies of strains SJ01 (yeast), SJ02 (Agg-1), and BJCA001A (Agg-2) by differential interference contrast (DIC) microscopy (**Fig 1B**) and scanning electron microscopy (SEM) (**Fig 1C)**. Strain SJ01 contained only single yeast-form cells of *C*. *auris*, while strains SJ02 and BJCA001A both formed clear multicellular aggregates. Interestingly, the SEM assays indicated that BJCA001A and SJ02 formed obviously distinct aggregating morphologies (**Fig 1C**). The cells of strain BJCA001A appeared unable to undergo physical separation during the cell division process leading to the formation of “clumps”, whereas the cells of strain SJ02 were able to be separated and appeared only to adhere to one another through cell-cell interactions. To further distinguish between these two aggregating morphologies of *C*. *auris*, we performed additional SEM assays. As shown in **Fig 1C**, the mother and daughter cells of strain BJCA001A, but not SJ02, exhibited obvious cell wall division defects.

To degrade extracellular proteins, including adhesins, we next treated the cells of strains SJ01, SJ02, and BJCA001A with proteinase K and trypsin. After treatment with either enzyme, the cell clumps of strain SJ02 separated into single yeast-form cells, while no obvious changes were observed in the aggregating morphologies of strain BJCA001A (**Fig 1B**). These results indicate that the two aggregating morphologies of *C*. *auris* strains SJ02 and BJCA001A are due to different mechanisms. Specifically, the aggregating form of strain SJ02 represents a distinct multicellular morphology resulting from cell surface protein-mediated cell-cell adhesion.

**Fig 2:**
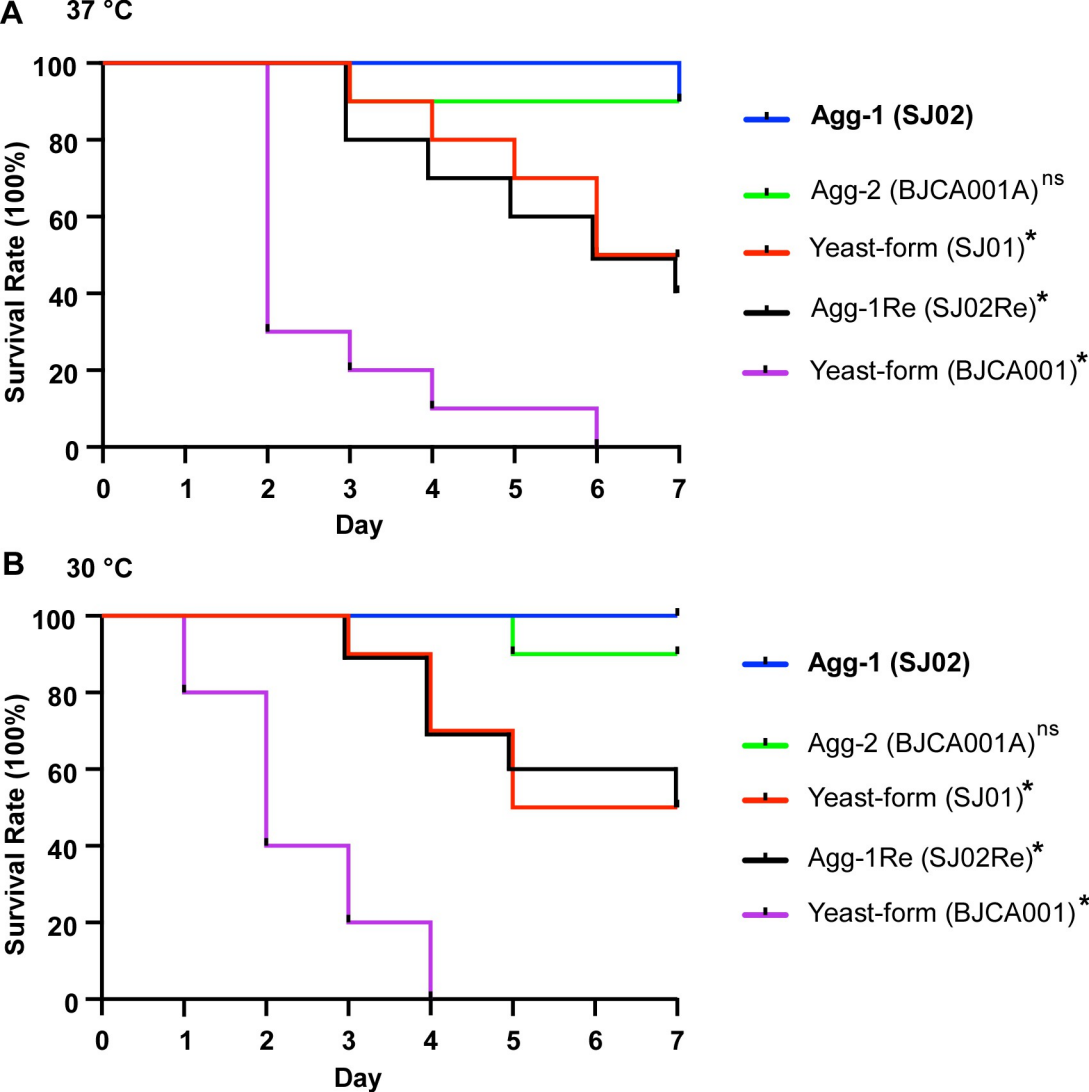
Survival curves of *G*. *mellonella* larvae infected with *C*. *auris* nonaggregative/yeast-form (SJ01 and BJCA001) and aggregative-form (SJ02 and BJCA001A) strains. Approximately 1 x 10^6^ *C*. *auris* cells of each strain were injected into each *G*. *mellonella* larva. Ten larvae were injected for each strain. ns, no significant difference; *, significant difference (p<0.05, by log rank test. Strain SJ02 served as the control). (A) Survival curves from experiments performed at 37°C. (B) Survival curves from experiments performed at 30°C.

Since cell aggregation has known effects on virulence of *C*. *auris* strains, we next evaluated the virulence of strains SJ01 (yeast-form), SJ02 (Agg-1), SJ02RE (yeast-form), BJCA001 (yeast), and BJCA001A (agg-2) using a *Galleria mellonella* infection model. As shown in **Fig 2**, compared to the yeast-form cells of SJ01, SJ02RE, and BJCA001, the two aggregating forms of SJ02 and BJCA001A both exhibited decreased virulence in the model. This attenuated virulence observed for these two strains could be due to a reduction in cellular dissemination in the *G*. *mellonella* larval bodies, which is a consequence of enhanced aggregation.

### Aggregative-form to nonaggregative/yeast-form transition in *C*. *auris* strain SJ02

**Fig 3:**
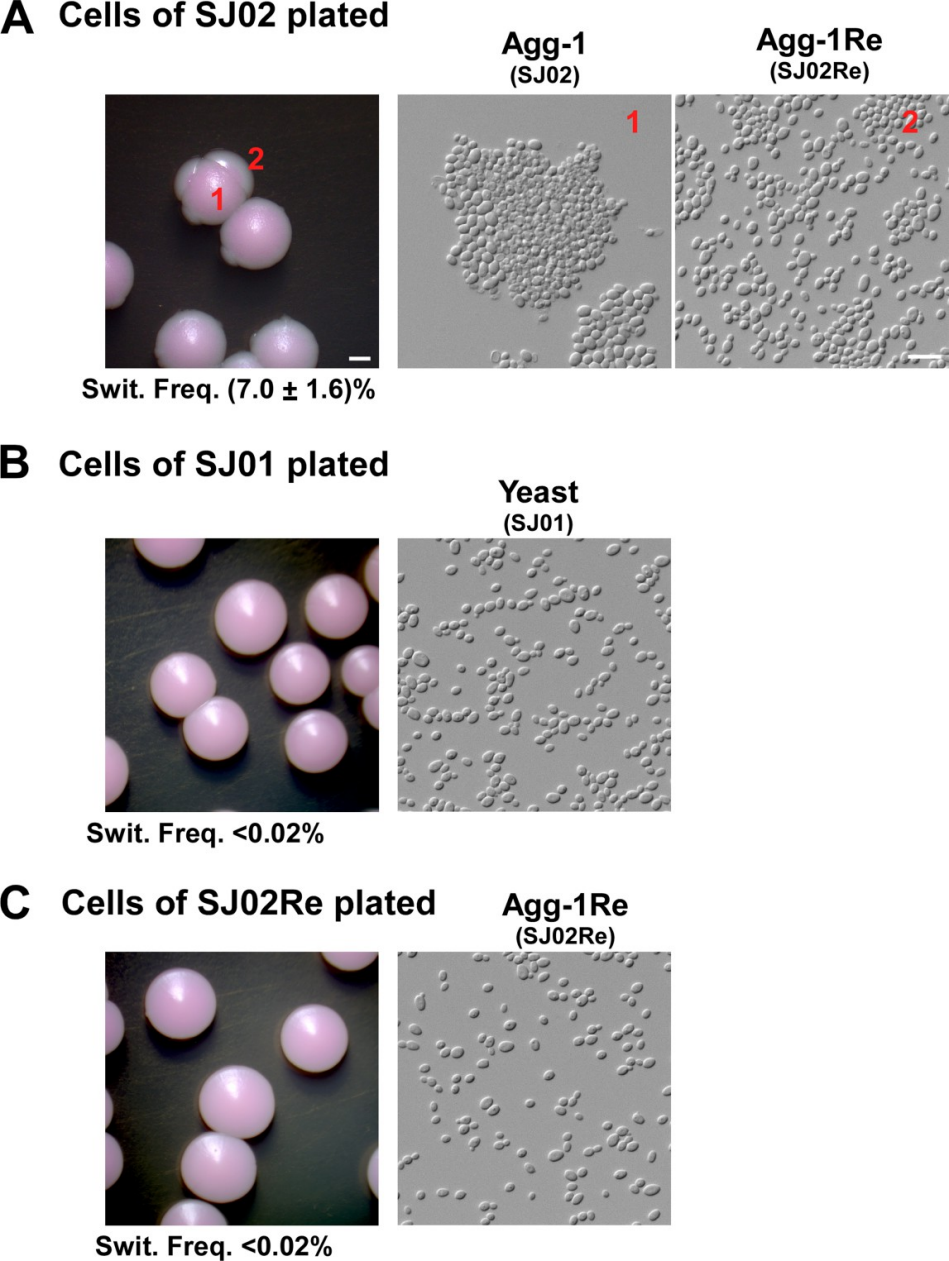
Spontaneous switching between the aggregating form and the single-celled nonaggregative/yeast-form. *C*. *auris* cells of the original aggregating form (Agg-1, SJ02), SJ01 nonaggregative/yeast-form, or SJ02Re nonaggregative/yeast-form were plated onto solid YPD medium containing 5 μg/mL phloxine B and cultured at 30°C for 5 days. (A) Colony and cellular morphologies of the aggregating form (Agg-1, SJ02) and spontaneously generated nonaggregative/yeast-form (sectors, Agg-1Re or SJ02Re). (B and C) Colony and cellular morphologies of the nonaggregative/yeast-form strains (SJ01) and (SJ02Re). Switch frequencies to the alternative form were shown below the corresponding colony images. <0.02% indicates no alternative morphological cells observed. Scale bar for cells, 10 μm; scale bar for colonies, 1 mm.

Interestingly, when grown on solid media for an extended culture period (≥5 days), some colonies of the aggregative-form strain SJ02 formed sectored colonies. When observed by microscopy, cells from those sectors were in the single-celled nonaggregating phenotype (i.e., they “returned” to the nonaggregative/yeast-form, strain SJ02Re, **Fig 3A**), suggesting that the aggregating phenotype of strain SJ02 is able to switch to the nonaggregative-form at a high frequency (~7%). However, no nonaggregative-form to aggregative-form switching was observed in either strain SJ01 or the “returned” nonaggregative-form strain SJ02Re (frequency < 0.02%, **Fig 3B** and **3C**).

As demonstrated in **Fig 1**, the “returned” nonaggregative-form strain SJ02Re and clinical strain XM03-1 [19] exhibited similar morphologies to those of strain SJ01.

### Comparative analysis of biofilm formation in the nonaggregative/yeast-form and aggregative-form strains of *C*. *auris*

**Fig 4:**
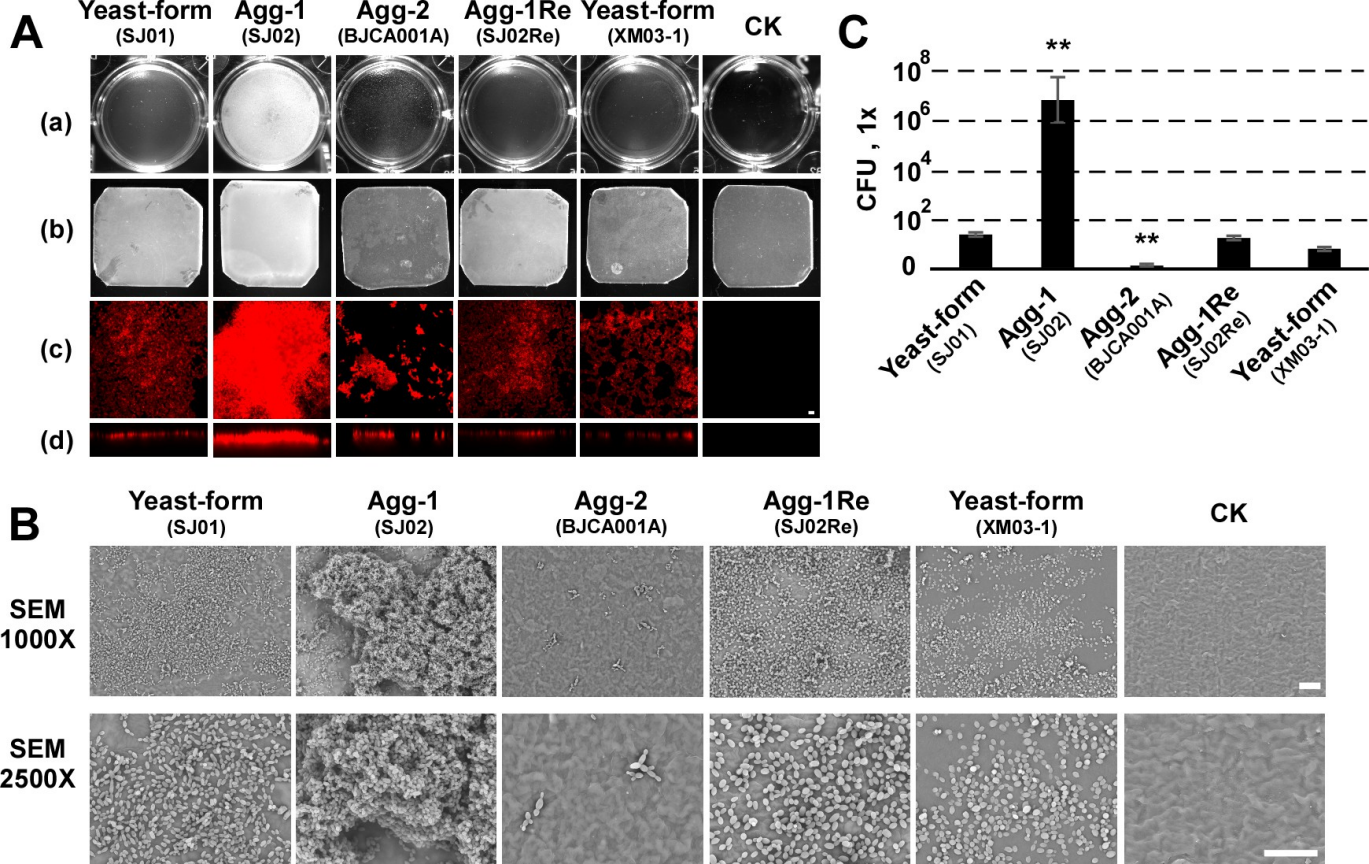
Biofilm development of the nonaggregative/yeast-form (SJ01), Agg-1 (SJ02), Agg-2 (BJCA001A), Agg-1Re (SJ02Re), and XM03-1 strains of *C*. *auris*. The same set of strains were used as in **Fig 1**. CK, no fungal cells added. (A) Biofilm formation on the bottoms of 24-well plate polystyrene plates (a) or on silicone squares (b, c, and d). *C*. *auris* cells were incubated in RPIM1640 medium for 48 hours with shaking at 37°C. A silicone square was placed in each well for the lower panel. The polystyrene plates or silicone squares were gently washed with water and imaged. Biofilms formed on silicone squares were stained with calcofluor white for confocal microscopy assays with 405 nm laser excitation lines. CSLM top and side views were performed, respectively (c and d). Scale bar, 20 μm. (B) Low and high magnification SEM images of *C*. *auris* biofilms on silicone squares. Scale bar, 20 μm. (C) Quantitative analysis of cell numbers in the polystyrene plate well biofilms (panel A). The biofilms were washed with 1 x PBS and treated with proteinase K for 1 hour, resuspendend, and then diluted and plated on YPD medium. **p < 0.01, two-tailed Student *t* test.

Prior work has shown that the ability to form aggregates influences biofilm development in *C*. *auris* [4,7,10]. However, both promoting and inhibitory effects of cell aggregation on biofilm development have been reported [4,7,10]. We next tested the biofilm forming abilities of strains SJ01, SJ02, SJ02Re, BJCA001A, and XM03-1 to develop biofilms on polystyrene plates and silicone squares. Comparatively, strain SJ02 developed robust biofilms, while the typical aggregative-form strain (BJCA001A) developed weak biofilms on both polystyrene and silicone substrates (**Fig 4A**). The nonaggregative-form stains SJ01, SJ02Re, and XM03-1 exhibited comparable abilities to form biofilms, and were much weaker than biofilms formed by strain SJ02 (**Fig 4A**). Consistently, SEM microscopy assays performed on silicone squares indicated the same order of biofilm forming abilities of the three strains (SJ02 > SJ01 (SJ02Re or XM03-1) > BJCA001A, **Fig 4B**). To further evaluate the biofilms, we performed colony forming unit (CFU) assays on the biofilms formed on the bottoms of polystyrene plates, and verified the strong biofilm forming ability of strain SJ02 (**Fig 4C**). These findings correlate the new aggregating morphology of strain SJ02 with an enhanced capacity for biofilm development.

### Enhanced ability of skin colonization of the new aggregative-form strain of *C*. *auris*

**Fig 5:**
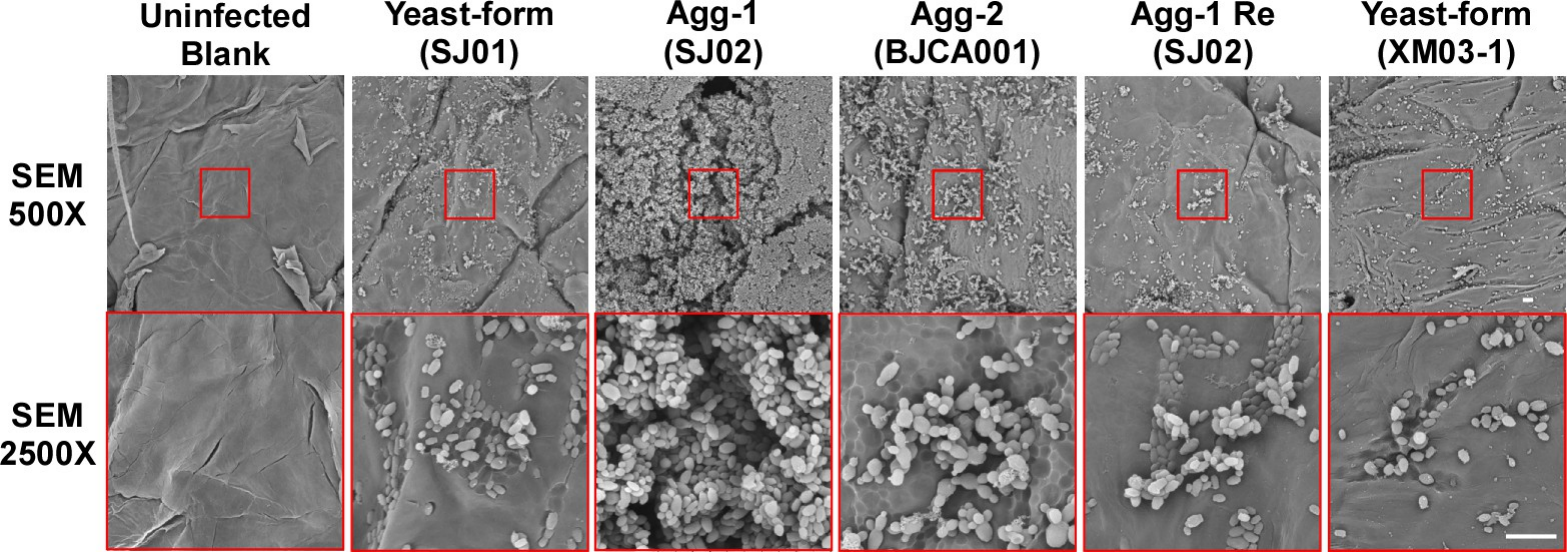
Skin infection assays using a newborn mouse model. *C*. *auris* strains: nonaggregative/yeast-form (SJ01), Agg-1 (SJ02), Agg-2 (BJCA001A), Agg-1Re (SJ02Re), and nonaggregative/yeast-form XM03-1. Approximately 5 x 10^6^ *C*. *auris* cells of each strain in 2 μL ddH_2_O were inoculated on the dorsal back skin of newborn mice. After the skin surface dried, a small sterilized glossy paper was affixed on the inoculated spot with medical tape. After 24 hours of infection, the infected skin areas were excised and gently washed with 1 x PBS. Low and high magnification SEM images are shown. Scale bar, 10 μm.

Since adhesion and biofilm formation are important features in the colonization and infection of host skin and tissues, we next performed skin colonization/infection experiments with strains SJ01, SJ02, SJ02Re, BJCA001A, and XM03-1 using a newborn mouse skin infection model. *C*. *auris* cells of each strain were inoculated on the back skin regions of newborn mice. After 24 hours of infection, the colonized skin regions were gently washed with 1 x PBS and excised for SEM assays. Consistent with the results of biofilm development, the aggregative-form strain SJ02 exhibited the most robust skin colonization ability and formed thick biofilm-like structures (**Fig 5**). The nonaggregative-form strains SJ01, SJ02Re, and XM03-1 demonstrated relatively weak abilities to colonize skin. Interestingly, strain BJCA001A showed an intermediate ability to colonize skin in this infection model and its colonization led to the formation of numerous small pits on the skin tissue. One possible explanation for this phenomenon is that strain BJCA001A belongs to the South Asian clade (clade I, *MTL***a**) and the other four strains belong to the South African clade (clade III, *MTL*α). We previously reported that *C*. *auris* strains of the South Asian clade secrete more secreted aspartyl proteases than strains of the South African clade at low temperatures [19]. Secreted aspartyl proteases could damage the skin and lead to the formation of small pits.

### Genomic analyses reveal that amplification of *ALS4* contributes to the aggregating phenotype of strain SJ02

To uncover the underlying mechanism of the formation of the new aggregating phenotype in *C*. *auris*, we sequenced the genomic DNA of strains SJ01, SJ02, SJ02Re, BJCA001A, and XM03-1 using next-generation sequencing approaches. The number of pairwise SNPs and associated mutated genes between strains SJ01, SJ02, and SJ02Re were fewer than those between strains isolated from different patients in China of the same genetic clade (**S1 Table**). Based on the biological roles of the involved genes, we did not find obvious mutations caused by SNPs or INDELs that were associated with the aggregating phenotypes (**S1 Table** and **S1 Dataset**).

**Fig 6:**
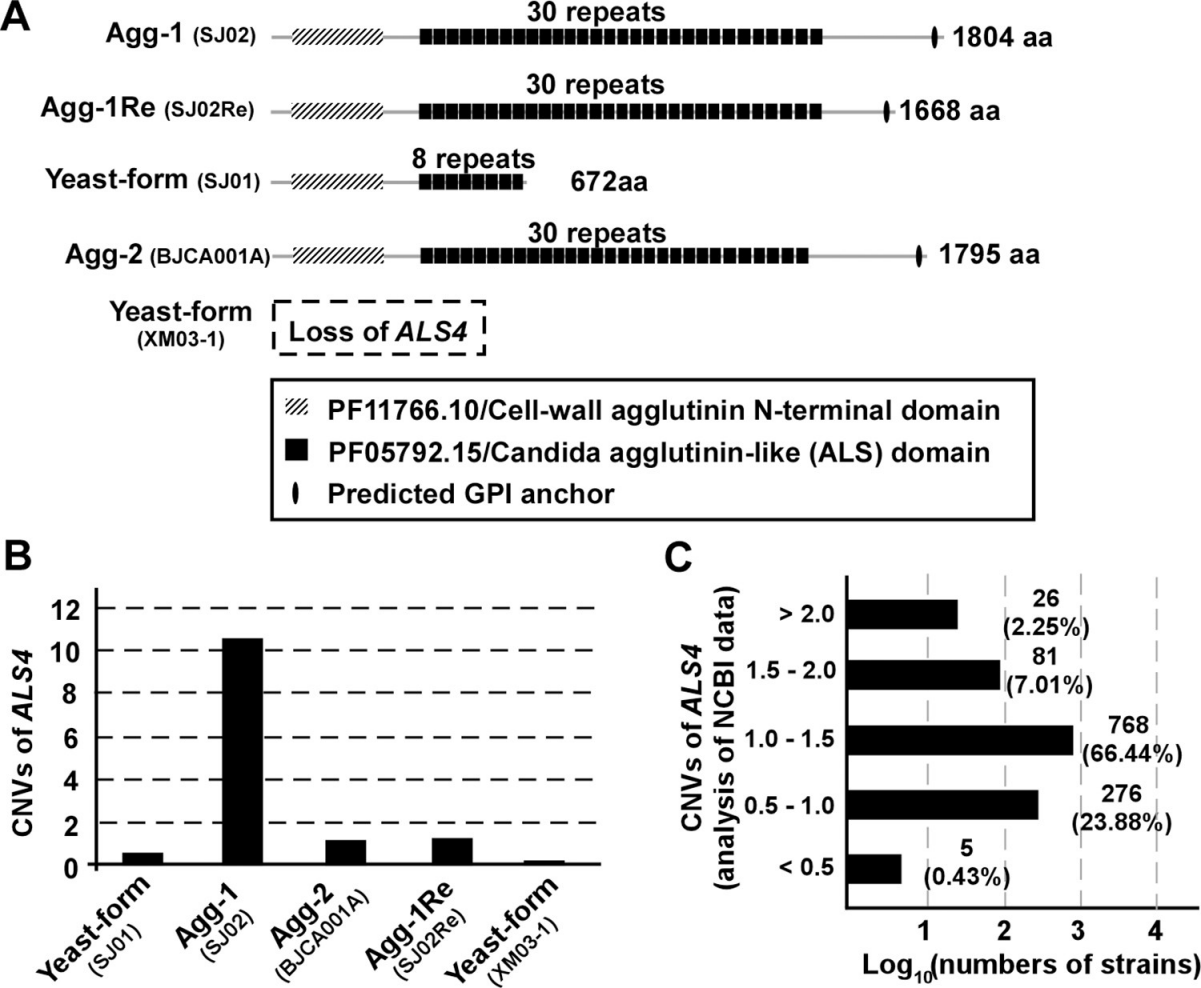
Protein sequence variations and CNVs of *ALS4* of *C*. *auris* clinical isolates. (A) Schematic diagrams of the predicted Als4 proteins of the nonaggregative/yeast-form (SJ01), Agg-1 (SJ02), Agg-2 (BJCA001A), Agg-1Re (SJ02Re), and nonaggregative/yeast-form XM03-1 strains. The nonaggregative/yeast-form strain (SJ01) carries a truncated *ALS4* gene, which encodes a protein containing only 8 repeats of the Als domain. The Xiamen isolate XM03-1 is a natural strain missing the *ALS4* gene. (B) Copy number variations (CNVs) of *ALS4* of the yeast-form (SJ01), Agg-1 (SJ02), Agg-2 (BJCA001A), Agg-1Re (SJ02Re), and yeast-form (XM03-1) strains. Both the nonaggregative/yeast-form (SJ01) and “returned” nonaggregative/yeast-form (SJ02Re) strains carry a single copy of the *ALS4* gene, whereas the aggregative-form strain (SJ02) carries 10 copies of the *ALS4* gene. (C) CNV analyses of *ALS4* for 1156 *C*. *auris* strains (genomic sequences were retrieved from the NCBI database).

We next performed copy number variation (CNV) analysis and found substantial amplification of the *ALS4* gene (10 copies) in strain SJ02 (**Fig 6**). For strain SJ02, we found that eight copies of *ALS4* were full-length and just two copies of *ALS4* were nearly full-length with ~460 bp missing at the 3’ end of the *ALS4* ORF. The *ALS* gene family encodes a set of cell wall glycoproteins in several pathogenic *Candida* species that are often involved in the regulation of adherence to surfaces and biofilm formation [14]. We speculated that the genomic amplification of *ALS4* contributed to the aggregating phenotype of *C*. *auris* strain SJ02. Consistent with our hypothesis, the nonaggregative-form strain SJ01 had a shortened copy of *ALS4*, and the “returned” nonaggregative-form strain SJ02Re and typical aggregative-form strain BJCA001A each carried a single copy of *ALS4* that was nearly full length (**Fig 6A** and **6B**). We note that since strain SJ01 does not carry a full-length copy of *ALS4*, it could not be the parental strain of isolate SJ02, which carries ten copies of full-length/nearly full-length *ALS4*. Interestingly, no sequence signal of *ALS4* was detected in strain XM03-1, suggesting that *ALS4* has been completely lost in this strain (**Fig 6A** and **6B**).

**Fig 7:**
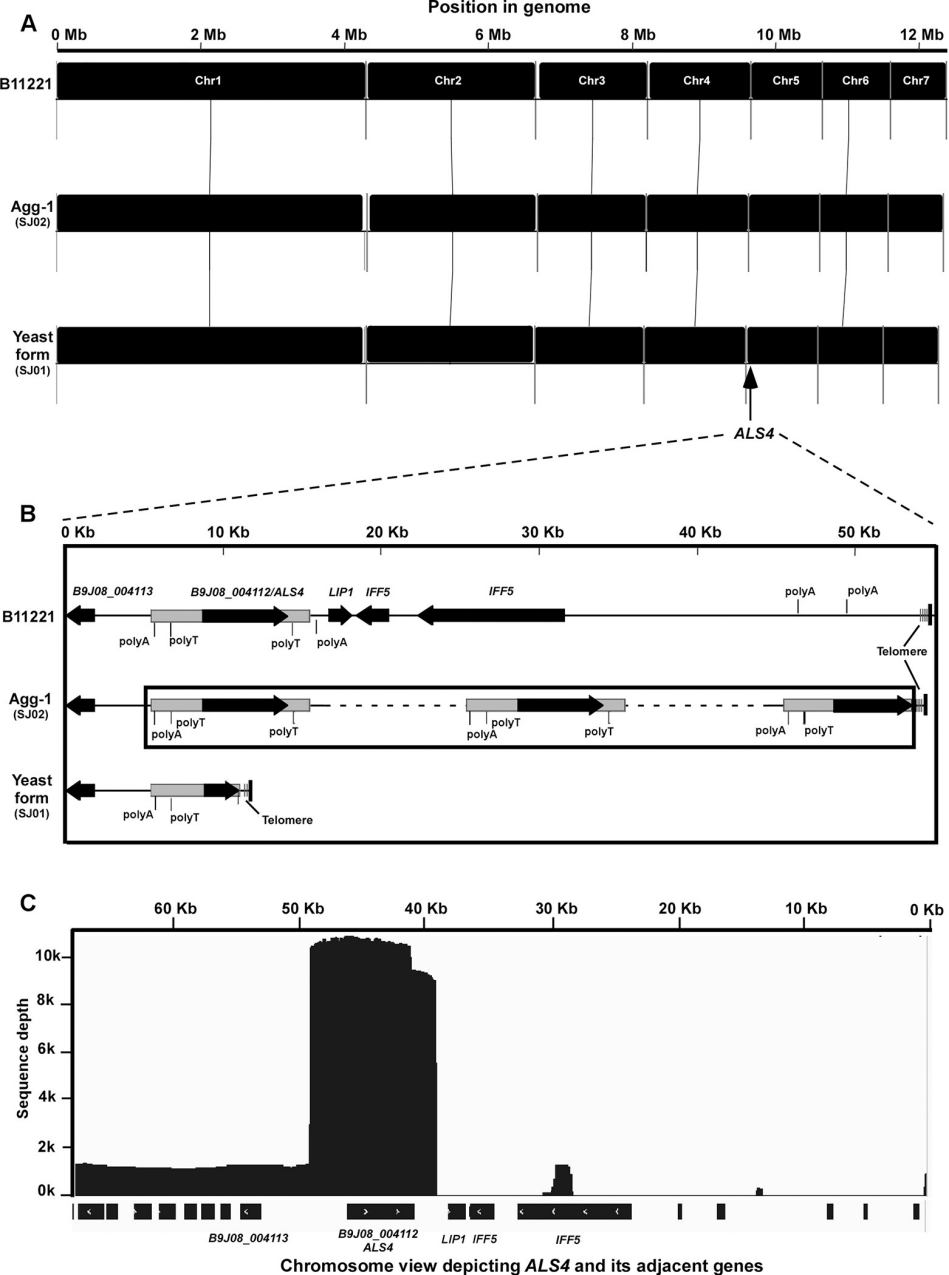
Genomic analysis of the nonaggregative/yeast-form (SJ01) and aggregative-form (SJ02) strains of *C*. *auris*. Long-read sequencing and data analyses were performed. (A) Genome synteny among strains SJ01, SJ02, and reference strain B11221. Black blocks indicate shared syntenic regions of the genomes. (B) Synteny schematic depicting the orientation and conservation of *ALS4* and its adjacent genes. The copy number was estimated using Illumina sequencing data. Tandem repeats of *ALS4* are indicated by dashed lines. (C) Sequence depth of *ALS4* and its adjacent region (estimated based on PacBio Sequel II sequencing data). The sequence depth was calculated by IGV.

To further compare the genomic organization of the nonaggregative-form strain SJ01 and aggregative-form stain SJ02, we sequenced the genomes of strains SJ01 and SJ02 using long-read sequencing and assembled their genomes. Similar genomic sizes (~12.4 Mb) and chromosome numbers (7) were observed for the two strains and their genomes exhibited overall high levels of synteny (**Fig 7**). Moreover, we observed that there were many polyA/polyT sequence motifs located adjacent (on both sides) of the *ALS4* repeat, possibly promoting the expansion of the *ALS4* gene. Overall, the long-read sequencing assays confirmed that the tandem-repeat amplification of *ALS4* contributed to the aggregative phenotype of strain SJ02. A comparative analysis of the genomes of strains SJ01, SJ02, and reference strain B11221 [20] is shown in **Fig 7 and S1 Dataset**.

The comparative genomic analysis demonstrated that a number of genes including *IFF5* and *LIP1*, adjacent to the *ALS4* locus, were missing in strains SJ01, SJ02, SJ02Re, and XM03-1 (possibly due to the instability of the subtelomeric region), but were present in strains BJCA001A and B11221 (**S2 Table**). *IFF5* encodes a GPI-anchored adhesin-like protein, and *LIP1* encodes a secreted lipase that is transcriptionally induced during biofilm development. Since these two genes were missing in the genomes of both strains SJ01 and SJ02, the absence of *IFF5* and *LIP1* could not be associated with the *ALS4*-mediated adherence and biofilm formation phenotypes observed in strain SJ02.

### RNA-Seq and quantitative transcriptional expression analyses

**Fig 8:**
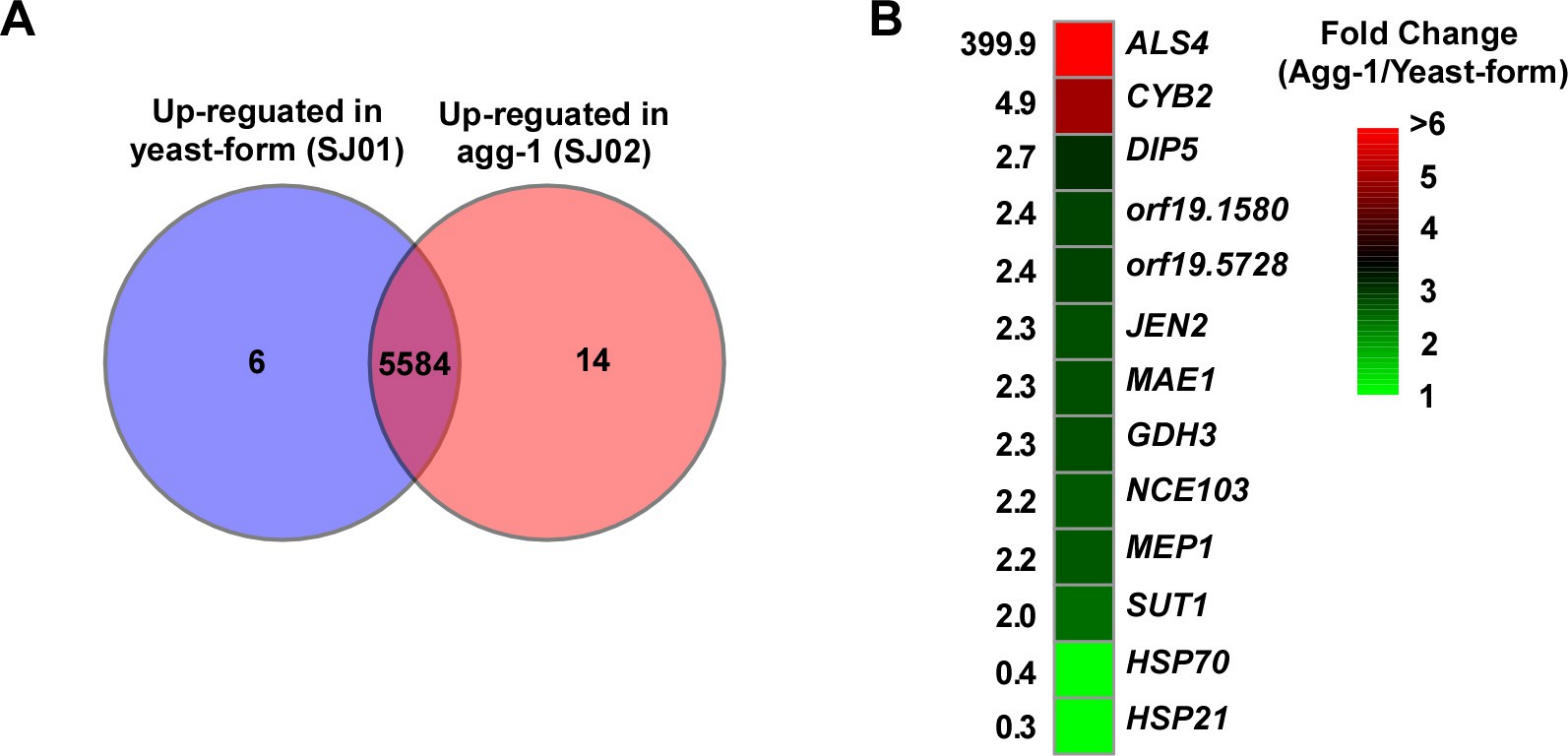
Global transcriptional profiles of the nonaggregative/yeast-form (SJ01) and aggregative-form (SJ02) strains of *C*. *auris* by RNA-Seq. (A) Venn diagram depicting differentially expressed genes. A twofold difference cut-off and false discovery rates (FDRs) less than 0.01 were used. (B) R package heatmap depicting the differentially expressed genes associated with biofilm development. Fold changes are shown.

To further confirm the relationship between *ALS4* and the aggregative phenotype, we performed transcriptomic analysis by RNA-seq for strains SJ01 and SJ02. As shown in **Fig 8A** and **S3 Dataset**, only 20 genes were found to be differentially expressed between the nonaggregative-form and aggregative-form of strains SJ01 and SJ02 (using a twofold cutoff). Of them, 14 genes were upregulated in cells of strain SJ02 and 6 were upregulated in cells of strain SJ01. As expected, the relative expression level of *ALS4* in strain SJ02 was much higher than that of strain SJ01. The average FPKM value of *ALS4* in strain SJ02 was about 400 times higher than that of strain SJ01 (**Fig 8B**). Quantitative transcriptional expression assays verified that *ALS4* was highly expressed in strain SJ02, whereas the relative expression levels of *ALS4* in cells of SJ02Re and BJCA001A were comparable to that of cells of strain SJ01 (not shown). These results suggest that the genomic amplification of *ALS4* in *C*. *auris* is a likely reason for the increased aggregation and biofilm formation observed in strain SJ02.

Other than *ALS4*, the majority of differentially expressed genes only showed a twofold to threefold difference in expression levels between the two strains. The orthologs of 12 other differentially expressed genes have been annotated to be associated with biofilm development in *C*. *albicans* (**Fig 8B** and **S2 Dataset**; http://www.candidagenome.org/). Eight genes are involved in metabolism processes, especially mitochondrial metabolism (e.g., *CYB2* and *MAE1*). Interestingly, the carbonic anhydrase-encoding gene *NCE103*, which is required for the conversion of CO_2_ to bicarbonate in fungi, was upregulated in cells of the aggregative-form strain SJ02. Aggregation could hinder CO_2_ diffusion in *C*. *auris* cells. Given the critical role of CO_2_ in the regulation of metabolism and morphological transitions, the increased expression of *NCE103* in the aggregated cells of *C*. *auris* was not unexpected.

**Fig 9:**
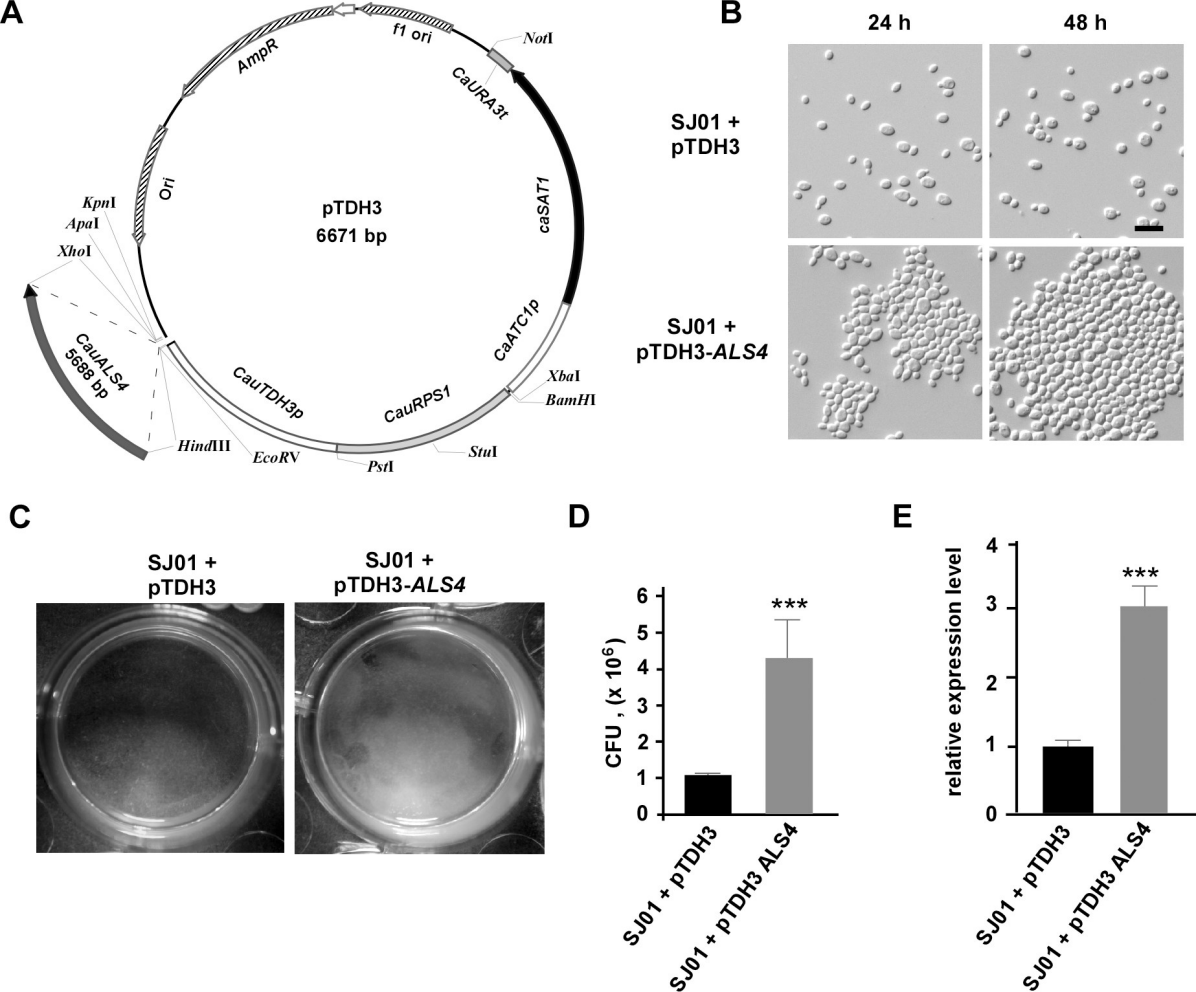
Ectopic expression of *ALS4* in strain SJ01 promotes cell aggregation and biofilm formation. (A) Diagram of plasmid pTDH3-ALS4. *RPS1* and TDH3-associated fragments were amplified from genomic DNA of *C*. *auris*. c*aSAT1*, *CaACT1* promoter (*C*. *albicans ACT1p*), and *CaURA3* terminator (*C*. *albicans URA3t*) were amplified from plasmid pNIM1. (B) Cellular morphologies of the control and ALS4-ecotopic strains. Cells of strain SJ01 (bearing a shortened single copy of *ALS4*) were transformed with linearized plasmids pTDH3 (vector-alone control) or pTDH3-ALS4. Fungal cells were cultured in liquid YPD medium at 30°C for 24 or 48 hours. Scale bar, 10 μm. (C) Biofilm formation of the control and *ALS4*-ecotopic strains on the bottoms of 24-well plate polystyrene plates. (D) Quantitative analysis of CFUs obtained from biofilms formed on the bottoms of the polystyrene plate wells in D. ***p < 0.001, two-tailed Student’s *t* test. (E) Relative expression levels of *ALS4* in the control and ALS4-ecotopic strains determined by Q-RT-PCR assays. ***p < 0.001, two-tailed Student’s *t* test.

To verify the promoting role of *ALS4* in the adherence and biofilm formation in *C*. *auris*, we constructed an ectopic expression plasmid pTDH3-ALS4 (**Fig 9A**). As predicted, ectopic expression of *ALS4* under the strong *TDH3* promoter in strain SJ01 notably enhanced the formation of cell aggregates and biofilms (**Fig 9B and 9C**). The enhanced capacity of biofilm formation was confirmed by quantifying *C*. *auris* CFUs obtained from the biofilms formed on the bottoms of 24-well plates (**Fig 9D**). Consistently, quantitative real-time PCR (Q-RT-PCR) assays demonstrated that the relative expression level of *ALS4* in the *ALS4-*ectopic strain was significantly higher than that of the control strain (**Fig 9E**). Together, these results confirm the role of Als4 in adherence and biofilm formation in *C*. *auris*.

### Copy number variation of *ALS4* is common across *C*. *auris* clinical isolates

As mentioned above, we isolated a *C*. *auris* strain (XM03-1, **Figs 1** and **6A** and **S2 Table**) from a patient with candidemia that lost *ALS4*, supporting the instability at this locus. To support the idea that copy number variation of *ALS4* is common in clinical isolates, we analyzed the genome sequences of 1156 *C*. *auris* strains available in the NCBI database (https://www.ncbi.nlm.nih.gov/). We found that 26 strains carried more than two copies of *ALS4* (2.3%, CNV ≥ 2), 81 strains carried 1.5–2.0 copies (7%, 1.5≤ CNV< 2), and 5 strains carried less than 0.5 copies of *ALS4* (0.4%, CNV ≤ 0.5, **Fig 6C** and **S2 Dataset**). These findings provide additional support that *ALS4* copy number variation is common among *C*. *auris* clinical isolates.

**Fig 10:**
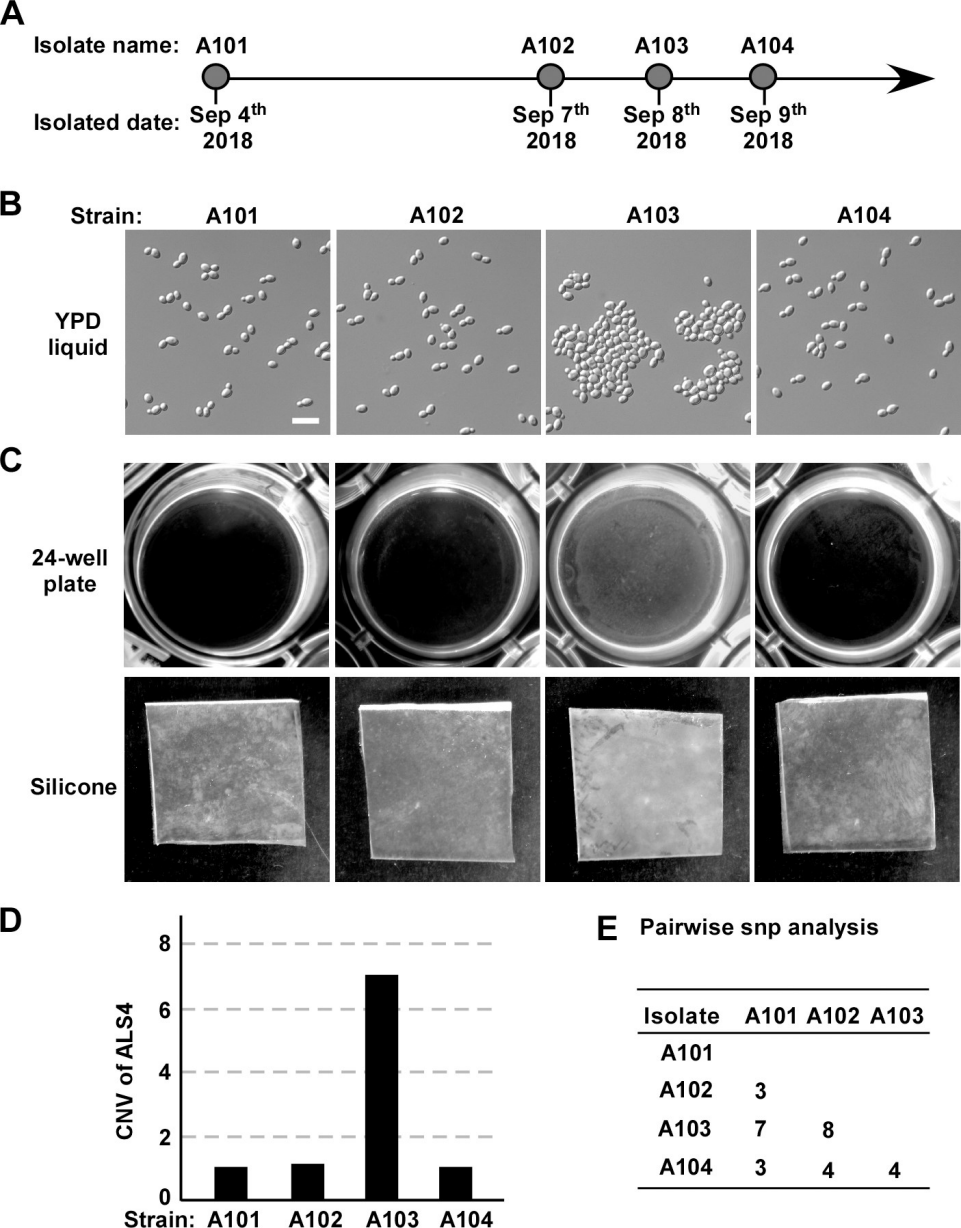
Identification of *C*. *auris* strain A103 with enhanced adherence and biofilm formation. (A) Four *C*. *auris* strains were isolated at different time points from the same patient. (B) Cellular morphologies of strains A101, A102, A103, and A104. *C*. *auris* cells were cultured in liquid YPD medium at 30°C for 24 hours. Scale bar, 10 μm. (C) Biofilm formation of the four *C*. *auris* strains on the bottoms of 24-well polystyrene plates and silicone squares. *C*. *auris* cells were incubated in RPIM1640 medium for 48 hours with shaking at 37°C. (D) Copy number variations (CNVs) of *ALS4* of the four isolates. SRA database accession numbers for the genomic sequences: A101 (SRR9316791), A102 (SRR9316792), A103 (SRR9316793), and A104 (SRR9316794). (E) Pairwise SNP analysis among the four clinical strains.

We and our collaborators recently published a report on a *C*. *auris* outbreak in a general hospital in China [21]. We revisited the phenotypes of the associated *C*. *auris* strains from this recent study and found another clinical isolate exhibiting enhanced adherence. As shown in **Fig 10A**, four strains (A101, A102, A103, and A104) were isolated from urine samples of the same patient at different time points. Similar to strain SJ02, strain A103 showed an aggregating phenotype and formed enhanced biofilms on both the bottoms of 24-well plates and silicone squares (**Fig 10B** and **10C**). Genomic analysis demonstrated that strain A103 carries seven copies of *ALS4*, while the other three strains contain a single copy of *ALS4* (**Fig 10D**). We further found that there were only three to eight SNP differences among the four isolates, suggesting that these strains originated from the same ancestor (**Fig 10E**). Taken together, our findings indicate that the *ALS4* locus is unstable in *C*. *auris* clinical isolates and is able to undergo copy number changes *in vivo*.

## Discussion

Adhesion is important not only for the development of multicellular aggregations but also for pathogen-host interactions. In pathogenic *Candida* species, the Als proteins are large cell-surface glycoproteins that play critical roles in the processes of adhesion to host and abiotic surfaces, aggregative behaviors, and biofilm development [14,15,22]. In the present study, we investigated variations of the *ALS4* gene in *C*. *auris* clinical isolates and identified two clinical strains (SJ02 and A103) with a new aggregative phenotype due to enhanced adherence of adjacent cells. This aggregative morphology differs from the previously reported aggregative phenotype [7], which is caused by cell division defects. This new aggregative morphology, is not the result of cell division defects and plays roles in the adherence of *C*. *auris* to host and abiotic surfaces, and in virulence in the *G*. *mellonella* infection model (**Figs 1**,**2**,**4**,**5**,**9**, and **10**). Genomic analyses indicated that amplification of *ALS4* conferred the strains SJ02 and A103 with an enhanced ability to adhere to surfaces and form biofilms. Unlike *C*. *albicans*, which is commensal to the gastrointestinal and genitourinary tracts, *C*. *auris* is considered to be a largely skin colonizer [23]. Since Als4 is a cell surface adhesin, the amplification of *ALS4* could be important in both the commensal and pathogenic life-styles of *C*. *auris*.

There are nine members of the Als family (Als1-9) in *C*. *albicans*, and the *ALS* genes display variability in transcriptional regulation, gene size, copy numbers, and additional coding regions [14]. Only three *ALS* orthologs (B9J08_004112/*ALS4*, B9J08_002582, and B9J08_004498) have been found in *C*. *auris*, all of which are orthologs to *C*. *albicans ALS4*. In the present study, we focused on the biological function of *C*. *auris* B9J08_004112/*ALS4*, which exhibits a high variation in copy number in *C*. *auris* clinical isolates (**Fig 6** and **S2 Dataset**).

We collected five *C*. *auris* isolates (SJ01, SJ02, SJ02Re, BJCA001A, and XM03-1) and performed next-generation and long-read genome sequencing experiments. We found that the *ALS4* gene exhibits obvious copy number or sequence variability in the five isolates. Strains SJ01 and SJ02 were isolated from the same patient with a long-term urinary tract infection, and strain XM03-1 was isolated simultaneously from a patient with a *C*. *auris* bloodstream infection [19]. SJ02Re and BJCA001A were derivatives of strains SJ02 and BJCA001 [18], respectively. Strain SJ01 bears a truncated version of *ALS4*, whereas strain SJ02 carries ten tandem copies of the full-length gene. Based on comparative genomic and phylogenetic analyses, SJ01 and SJ02 appear to be two isolates that recently diverged from a common ancestor. The “returned” nonaggregative/yeast-form strain SJ02Re carries a slightly shortened version of *ALS4*, strain BJCA001A contains a nearly full-length version of *ALS4*, and *ALS4* was completely missing in strain XM03-1 (**Fig 6A**). Copy number variation analysis of the genomic sequences available in the NCBI database verified that the *ALS4* gene is highly variable among *C*. *auris* clinical isolates (**Fig 6** and **S2 Dataset**). A revisit of the biological and genomic features of another clinical isolate (A103) that was recently published in a *C*. *auris* outbreak in China revealed that like SJ02, A103 also exhibits similarly enhanced adherence and biofilm formation abilities. We found that A103 contains seven copies of *ALS4* (**Fig 10**). These results suggest that the variation of the *ALS4* locus is common among clinical isolates and that this subtelomeric region may be subject to rapid change in *C*. *auris*. In many other fungi, subtelomeric regions tend to exhibit a high rate of rearrangement and recombination, such as, for example, in the *FLO* genes of *S*. *cerevisiae* [17] and the *EPA* genes of *C*. *glabrata* [24]. Another example is the tandem amplification of the telomere-adjacent gene *ARR3*, which encodes an arsenite efflux transporter in *Cryptococcus neoformans* [25]. The subtelomeric regions of eukaryotic organisms are often evolutionarily active and contain repetitive DNA sequences. Genomic rearrangements occur frequently in these regions due to high rates of recombination or double-strand breaks, which cause genomic instability. For example, the *FLO* genes at the subtelomeric region in *S*. *cerevisiae* exhibit a high frequency of intergenic recombination [26,27]. The loss and gain of *ALS4* in *C*. *auris* could be due to a similar mechanism to that observed for *S*. *cerevisiae*. Moreover, the tandem repeat sequences of *ALS4* in *C*. *auris* could also facilitate recombination and rearrangement of the subtelomeric region. Together, microevolution of the subtelomeric regions may represent a general strategy of fungi to adapt to rapidly changing environments.

Given the adherence and hydrophobic characteristics of Als4, it is reasonable that the amplification of the *ALS4* gene in *C*. *auris* results in enhanced biofilm development and skin colonization. The transcriptional expression level of *ALS4* has been further amplified in the strain SJ02, perhaps due to the expansion of copies of *ALS4* to a relatively distant region of the telomere. The repressing effect caused by subtelomeric epigenetic factors could be nullified in the *ALS4* copies that are telomere distant. Global transcriptional expression analyses demonstrated that only 20 genes including *ALS4* showed a twofold or greater change in expression between the nonaggregative/yeast-form strain SJ01 and aggregative-form strain SJ02 (**Fig 8 and S2 Dataset**). Of these differentially expressed genes, several are annotated to be involved in the regulation of biofilm development in *C*. *albicans*. These results suggest that the amplification of *ALS4* in the subtelomeric region has a limited overall impact on the global transcriptional expression profiles of *C*. *auris*, but dramatically increases the expression of the Als4 adhesin, thus enhancing adherence and biofilm development.

In this study, we demonstrate that there are two distinct types of aggregating phenotypes in *C*. *auris*. The typical aggregative phenotype is due to a defect in cell division and in the release of budding daughter cells and has been reported by several other groups in prior studies. The other new aggregative phenotype that we report here is due to increased cell-cell adhesion likely resulting from the expansion of the adhesin gene *ALS4*. Overall, our findings provide new insights into the understanding of the biology and virulence features of this important emerging human fungal pathogen. However, many open questions remain to be addressed. For example, does the rapid microevolution of the *ALS4* locus contribute to the high transmission capacity of *C*. *auris* within healthcare settings? Is the variation of Als4 associated with the persistence of the fungus on skin and environmental surfaces, thus increasing the potential for hospital outbreaks? How did amplification of *ALS4* occur? Does the host environment affect genomic stability of *C*. *auris*?

## Materials and methods

### Ethics statement

All animal experiments were performed according to the guidelines approved by the Animal Care and Use Committee at Fudan University. The present study was approved by the Committee.

### *C*. *auris* isolates and culture conditions

Clinical strains SJ01 (nonaggregative/yeast-form) and SJ02 (Agg-1, aggregative-form) were isolated simultaneously from the urine of a hospitalized patient in the Shengjing Hospital of China Medical University (Shenyang, China). Clinical strain XM03-1 was isolated from the first affiliated hospital of Xiamen University [19]. Strain BJCA001A (Agg-2, aggregative-form) was isolated from the mouse spleen following a systemic infection [6]. Strains A101, A102, A103, and A104 were isolated from urine samples of the same patient in a general hospital in China [21]. YPD medium (10 g/L yeast extract, 20 g/L peptone, and 20 g/L glucose), a rich medium, was used for routine growth of *C*. *auris*. To distinguish the colony morphology of nonaggregating/yeast-form and aggregating-form cells, phloxine B (5 μg/mL) was added to YPD medium.

### Whole genome sequencing and analysis

Single colonies of *C*. *auris* strains were inoculated into liquid YPD medium and grown at 30°C for 24 h. Fungal cells were collected, and genomic DNA was extracted using the TIANamp Yeast DNA Kit (TianGen Biotech, Beijing, China) according to the manufacturer’s protocol. Whole genome sequencing assays were performed by Berry Genomics Co., Beijing, China. The methods for library construction, genomic sequencing, and SNP and INDEL analyses were based on our previous publication [28]. The clean reads were mapped to the genomic assembly of *C*. *auris* strain B11221 (NCBI accession number: GCF_002775015.1) using BWA mem 0.7.17 software with default settings [29]. SAMTools v1.361 [30], Picard Tools v1.56 (http://picard.source-forge.net), and GATK v2.7.2 were used for variation analyses [31,32]. The sorted BAM datasets were used for copy number variation (CNV) analyses of all coding genes with command “samtools depth” across certain gene regions.

Single molecule real-time sequencing was performed by Beijing Novogene Bioinformatics Technology Co., Ltd. (Beijing, China) using the PacBio Biosciences (PacBio) Sequel small-molecule real-time sequencing system (Pacific Biosciences, Menlo Park, CA, USA). The sequence data generated from the Illumina platform were used to proofread the PacBio assembly sequence. The PacBio sequence data were error-corrected, trimmed, assembled, and scaffolded using Canu v1.8 software guided by a genome size of 12.5 Mb [33]. Quast v5.0.2 was used to determine the quality of the sixteen newly assembled genomes [34]. AUGUSTUS v3.2.1 was used to predict proteins [35]. Putative ORFs were searched against the NR database of NCBI (http://www.ftp.ncbi.nlm.nih) and the Candida genome database (http://www.candidagenome.org/).

For the assembly of the *ALS4* tandem structure and its adjacent region, PacBio long reads were first mapped to the genomic assembly of *C*. *auris* strain B11221 using the minimap2 v2.17-r941 software [36] with default settings. The sequence depth were analyzed with IGV v2.12.3 [37]. For the *ALS4* insert signal analysis, PacBio reads containing the *ALS4* repeat fragment were firstly extracted and re-mapped to the B11221 genome using minimap2 with parameter “—secondary = yes and -N 5” allowing for at least five secondary alignments.

### Q-RT-PCR and RNAseq experiments

Q-RT-PCR and RNAseq experiments were performed as described previously with slight modifications [28]. Single colonies of *C*. *auris* strains were inoculated and grown in liquid YPD medium at 30°C until they reached exponential growth. Cells were harvested for total RNA extraction. 1 μg of total RNA per sample was used to synthesize cDNA with RevertAid H Minus Reverse Transcriptase (Thermo Scientific, Inc., Beijing, China). Quantification of transcripts for Q-RT-PCR was performed using a Bio-Rad CFX96 real-time PCR detection system with SYBR green. The values were normalized to the *C*. *auris ACT1* gene. Three biological replicates were used. The following primers used were for PCR amplification:

C. auris-pACT1-RT-F:5-ATGTCAACATTCAGGCTGTC-3

C. auris-pACT1-RT-R:5-AGTAGTCAGTCAAGTCTCTTCC-3

C. auris B9J08_004112 (ALS4) RT-F:5-TAACTTGGAAGCCGCTGGTG-3

C. auris B9J08_004112 (ALS4) RT-R:5-TACCGTACTGACCATCATAT-3.

For the RNA-Seq analysis, total RNA was sequenced using an Illumina NovaSeq 6000 instrument (Berry Genomics Co., Beijing). Clean reads were aligned to the previously established *C*. *auris* assembly from NCBI (GCA_002759435.2) using HiSat2 v2.0.5. Transcript expression levels were estimated with Stringtie v1.3.3b [38]. Differentially expressed genes were analyzed using the DESeq2 package for R [39].

### Protease treatment assays

The nonaggregative/yeast-form or aggregative-form cells of *C*. *auris* were incubated at 30°C for 24 h. Cells were harvested and washed 3 times with 1 x PBS. Fungal cells were resuspended in 1 mL 1 x PBS and then treated with ddH_2_O, proteinase K (at a working concentration of 50 μg/mL) (TianGen Biotech, Beijing, China), or trypsin (at a working concentration of 0.25% (Gibico, Inc., Beijing) at 37°C for 1 h.

### Biofilm assays

Biofilm assays were performed as described previously with slight modifications [40,41]. Silicone squares (Cardiovascular Instruments Corp, PR72034-060N) and 24-well flat-bottom polystyrene plates (BD Falcon) were used for the biofilm assays. Overnight cultures of each strain in YPD were harvested, washed, and inoculated into fresh RPMI1640 liquid medium (OD_600_ = 0.5) for biofilm growth. The cultures were incubated at 37°C for 90 min at 200 rpm with agitation for initial adhesion. The polystyrene plate bottoms or silicone squares were gently washed with 1 x PBS and fresh RPMI1640 medium was added for an additional 24 h of growth at 37°C. Biofilms formed in the 24-well polystyrene plates were used for CFU assays. The biofilms were washed with 1 x PBS, treated with proteinase K for 1 hour, resuspended, and then diluted and plated on YPD medium. Fungal cells were cultured at 37°C for two days. Biofilms of each strains on silicone square were stained with calcofluor white and Fluor 405 nm for confocal scanning laser microscopy (CSLM) visualization. Confocal sections were acquired every 0.5 μm up to a total Z-stack thickness of 60 μm in sequential mode with lasers. CSLM top and side views were constructed.

### Scanning electron microscopy (SEM) assays

SEM assays were performed as described previously [40]. Fungal cells in exponential growth phase or on silicone squares were fixed with 2.5% glutaraldehyde and washed three times with 1 x PBS. The samples were then dehydrated with ethanol (with gradually increasing concentrations: 50%, 70%, 85%, 95%, and 100%), dried, and coated with gold.

### *Galleria mellonella* infection model

Larvae of *G*. *mellonella* (0.3–0.4 g) were purchased from Tianjin Huiyu biological technology Co. LTD. (Tianjin, China). Cells of *C*. *auris* were cultured on YPD medium at 30°C for 24 hours, collected, and washed twice with 1 x PBS. Approximately 1 x 10^6^ fungal cells in 10 μL 1 x PBS were injected into each larva as previously described [18,42]. After injection, the larvae were placed in plastic culture dishes and placed at 30°C or 37°C in the dark.

### Mouse skin infection virulence assays

All animal experiments were performed according to the guidelines approved by the Animal Care and Use Committee of the School of Life Sciences at Fudan University. Newborn BALB/c mice (aged 3–5 days) were used. Approximately 5 x 10^6^ cells of each *C*. *auris* strain in 2 μL ddH_2_O were inoculated onto the dorsal back skin of newborn mice. Small sterile filter papers were used to cover the inoculated areas and medical tape was used to affix the filter papers onto the inoculated areas once the water evaporated. After 24 hours of infection, the infected skin tissues were excised and fixed with 2.5% glutaraldehyde for SEM assays.

### Plasmid and *ALS4* ectopic expression strain construction

To construct the plasmid pTDH3 for gene ectopic expression in *C*. *auris*, we first subcloned a fusion PCR product of the *RPS1* locus (5’ and 3’) into the *Pst*I/*Bam*HI site of plasmid pBluescript II KS(+). Genomic DNA of *C*. *auris* strain BJCA001 was used as the template. A *Stu*I restrict enzyme site was introduced into the amplified fragment. Primers LT1704 (atatacCTGCAGAGGTCTCTTTAGAAACTCCATC), LT1705 (CTGTCTCTTGGTGAAAGCAAAGGCCTTGGCGAAAACTCTCAAAAC), LT1706 (GTTTTGAGAGTTTTCGCCAAGGCCTTTGCTTTCACCAAGAGAC AG), and LT1707 (atatacGGATCCTCGTTCACAGAACTTGTTTG) were used for PCR. A fragment of the *C*. *auris TDH3* promoter sequence was amplified from genomic DNA of *C*. *auris* strain BJCA001 with primers LT1710 (atatacCTGCAGAGGGAAAGAACTTGACATTCC) and LT1711 (atatacGATATCCATGTTGTATAAGGGAAAATAAA) and subsequently subcloned into the *Pst*I/*Eco*RV site of the plasmid. Then, the *caSAT1* cassette (*Candida*-adapted nourseothricin-resistant gene) was amplified from the plasmid pNIM1 [43] with primers cauSAT1F (atatacGCGGCCGC CGTCAAAACTAGAGAATAAT) and cauSAT1R (atatacTCTAGAGACCACCTTTGATTGTAAATAG) and subcloned into the *Xba*I/*Not*I site of the plasmid, generating plasmid pTDH3 (**Fig 9A**).

To ectopically express *ALS4* in *C*. *auris* strains XM03-1 and BJCA001, the coding region of *ALS4* was amplified from genomic DNA of strain BJCA001 by PCR using primers CauALS4F (5-ATTTTAAAGCTTATGAAACTTGCTTCGCTTGC-3) and CauALS4R (5-ATTTTACTCGAGTTAGAACATGAGGTGGAAAAGTGG-3). PCR products were digested with restrict enzymes *Hind*III and *Xho*I and then subcloned into the *Hind*III/*Xho*I site of plasmid pTDH3, generating plasmid pTDH3-ALS4. The plasmid pTDH3-ALS4 was linearized with *Stu*I. Linearized plasmids pTDH3-ALS4 and pTDH3, which served as a vector-alone control, were then transformed into XM03-1 and BJCA001.

### Statistical analyses

Two-tailed paired Student’s *t* tests and log rank tests were used for statistical analyses as stated in the figure legends.

## Supporting information

S1 Dataset SNPs, INDELs, and amino acid mutations between *C*. *auris* strains SJ01, SJ02, and SJ02Re. Sheet 1. Whole genomic SNP and INDEL comparisons between the nonaggregative/yeast-form strain (SJ01) and aggregative-form strain SJ02. Sheet 2. Amino acid mutation analysis between strains SJ01 and SJ02. Sheet 3. Whole genomic SNP and INDEL comparisons between the aggregative-form strain SJ02 and “returned” strain SJ02Re. Sheet 4. Amino acid mutation analysis between strains SJ02 and SJ02Re. In total, 38 SNPs and 90 INDELs were found between strains SJ01 and SJ02.
(XLSX)

S2 Dataset Copy number variation (CNV) analysis for *C*. *auris* genes between *C*. *auris* strains. Sheet 1. The nonaggregative/yeast-form (SJ01), aggregative-form Agg-1 (SJ02), “returned” nonaggregative/yeast-form Agg-1Re (SJ02), and aggregative-form BJCA001 strains. Sheet 2. CNV analysis of *ALS4* for 1156 *C*. *auris* strains. Sequences were retrieved from the NCBI database.
(XLSX)

S3 Dataset RNAseq analysis of the nonaggregative/yeast-form strain (SJ01) and aggregative-form strain SJ02. (XLSX)

S1 Table Pairwise SNPs and mutated proteins between different clinical isolates. (DOCX)

S2 Table Gene presence-absence polymorphisms at the subtelomeric region of chromosome 5. (DOCX)
