# Supplementary material for: Clinical isolates of Candida auris with enhanced adherence and biofilm formation due to genomic amplification of ALS4
Source: PLoS Pathog. 2023 Mar 13;19(3):e1011239. doi: 10.1371/journal.ppat.1011239 (PMC10035925; doi:10.1371/journal.ppat.1011239)
Supplement: S1 Table — (DOCX) [file ppat.1011239.s004.docx]

**S1 Table. Pairwise SNPs and mutated proteins between different clinical isolates.**

| **Number of pairwise SNPs （mutated amino acids）** | | | | | | | | |
| --- | --- | --- | --- | --- | --- | --- | --- | --- |
| **Samples** | **SJ02**  **(agg-1)** | **SJ02**  **(agg-1Re)** | **XM03-1** | **BJCA002** | **RICU2_A2** | **RICU9_A65** | **RICU7_A23** | **RICU10_A57** |
| **SJ01**  **(yeast-form)** | 38  (14) | 45  (15) | 91  (40) | 73  (37) | 80  (30) | 82  (36) | 78  (31) | 96  (35) |
| **SJ02**  **(agg-1)** | 0  (0) | 15  (3) | 67  (31) | 67  (28) | 56  (21) | 58  (27) | 54  (22) | 72  (26) |
| **SJ02**  **(agg-1Re)** | 15  (3) | 0  (0) | 64  (34) | 76  (31) | 53  (24) | 61  (30) | 51  (25) | 69  (29) |

Notes: All strains were collected in China. Accession numbers for genomic sequences: SRR17326416, SRR17326417, SRR17326415, SRR17326412, SRR17326411, SRR13425910, SRR9316744, SRR9316750, SRR9316781, and SRR9316775 for strains SJ01, SJ02 and SJ02Re, XM03-1^1^, BJCA002^2^, RICU2_A2, RICU7_A23, RICU9_A65, and RICU10_A57^3^. Pairwise SNPs and protein mutation variation analyses based on the genomic data. The numbers shown stand for SNPs (lower) and mutated proteins (upper) between the two compared strains.

**References：**

1. Bing J, Wang SJ, Xu HP, Fan SR, Du H, Nobile CJ, et al. (2021) A case of Candida auris candidemia in Xiamen, China, and a comparative analysis of clinical isolates in China. Mycology-an International Journal on Fungal Biology. doi: 10.1080/21501203.2021.1994479. PubMed PMID: 35186414; PubMed Central PMCID: PMC8856026.

2. Fan SR, Zhan P, Bing J, Jiang N, Huang YN, Chen DK, et al. (2021) A biological and genomic comparison of a drug-resistant and a drug-susceptible strain of Candida auris isolated from Beijing, China. Virulence 12: 1388-1399. doi: 10.1080/21505594.2021.1928410. PubMed PMID: 34060424; PubMed Central PMCID: PMC 8172162.

3. Tian SF, Bing J, Chu YZ, Chen JJ, Cheng ST, Wang QH, et al. (2021) Genomic epidemiology of Candida auris in a general hospital in Shenyang, China: a three-year surveillance study. Emerging Microbes & Infections 10: 1088-1096. doi: 10.1080/22221751.2021.1934557. PubMed PMID: 34027824; PubMed Central PMCID: PMC8183536.
