# Supplementary material for: Clinical isolates of Candida auris with enhanced adherence and biofilm formation due to genomic amplification of ALS4
Source: PLoS Pathog. 2023 Mar 13;19(3):e1011239. doi: 10.1371/journal.ppat.1011239 (PMC10035925; doi:10.1371/journal.ppat.1011239)
Supplement: S2 Table — (DOCX) [file ppat.1011239.s005.docx]

**S2 Table. Gene presence-absence polymorphisms at the subtelomeric region of chromosome 5.**

| ***C. auris* gene ID**  **(name)** | **Location on Chr. 5** | **Gene presence-absence polymorphism** | | | | | |
| --- | --- | --- | --- | --- | --- | --- | --- |
|  |  | **Yeast-form (SJ01)** | **Agg-1 (SJ02)** | **Agg-1Re (SJ02Re)** | **Yeast-form (XM03-1)** | **Agg-2 (BJCA001A)** | **B11221** |
| CJI97_004167 | 522-998 | X | X | X | X | X | √ |
| CJI97_004168  (TRX1) | 4660-5013 | X | X | X | X | √ | √ |
| CJI97_004169 | 7400-7882 | X | X | X | X | √ | √ |
| CJI97_004170 | 16021-16695 | X | X | X | X | √ | √ |
| CJI97_004171  (orf19.6382) | 19508-19819 | X | X | X | X | √ | √ |
| CJI97_004172  (IFF5) | 23449-32607 | X | X | X | X | √ | √ |
| CJI97_004173  (IFF5) | 34500-36488 | X | X | X | X | √ | √ |
| CJI97_004174  (LIP1) | 36751-38256 | X | X | X | X | √ | √ |
| **CJI97_004175**  **(B9J08_004112, ALS4)** | **40857-46271** | √ | √ | √ | X | √ | √ |
| CJI97_004176 | 53106-54839 | **√** | **√** | **√** | **√** | **√** | **√** |
| CJI97_004177  (orf19.2899) | 55626-56399 | **√** | **√** | **√** | **√** | **√** | **√** |
| CJI97_004178  (CPR6) | 56862-57956 | **√** | **√** | **√** | **√** | **√** | **√** |
| CJI97_004179  (CKA1) | 58327-59307 | **√** | **√** | **√** | **√** | **√** | **√** |
| CJI97_004180  (LTV1) | 59989-61359 | **√** | **√** | **√** | **√** | **√** | **√** |

**Notes**: Analysis was performed using samtools and an in-house script and used the genomic sequence of *C. auris* B11221 (<http://www.candidagenome.org/>) as the reference. CJI97_00**** stands for GeneBank accession number. √, gene presence; X, gene absence. Genomic sequence accession numbers: SRR17326416, SRR17326417, SRR17326415, SRR17326412, SRR17326413 and SRR3883453 for strains SJ01, SJ02 and SJ02Re, XM03-1, BJCA001A and B11221.

**Reference:**

1. Lockhart SR, Etienne KA, Vallabhaneni S, Farooqi J, Chowdhary A, Govender NP, et al. (2017) Simultaneous Emergence of Multidrug-Resistant Candida auris on 3 Continents Confirmed by Whole-Genome Sequencing and Epidemiological Analyses. Clin Infect Dis 64: 134-140. doi: 10.1093/cid/ciw691. PubMed PMID: 27988485; PubMed Central PMCID: PMC5215215.
